# Diversity in function and regulation of the Hippo pathway

**DOI:** 10.1186/2045-3701-3-34

**Published:** 2013-08-28

**Authors:** Dawang Zhou

**Affiliations:** 1 State Key Laboratory of Stress Cell Biology, School of Life Sciences, Xiamen University, Xiang’an District, Xiamen, Fujian 361102, China

## 

The Hippo pathway is recognized as an important regulator of tissue growth and cell fate [1-3]. Originally indentified in Drosophila, the Hippo pathway, also known as the Salvador–Warts–Hippo pathway, contains core kinases cascade, Hippo and Warts(Wts) coupled by the scaffold protein Salvador (Sav), as well as Mats. The activation of the Hippo pathway kinases results in phosphorylation and inactivation of the downstream transcriptional co-activator Yorkie which binds to the sequence-specific DNA-binding protein Scalloped and enhances the expression of proliferative and pro-survival genes. In general, the primary function of the Hippo signaling pathway is to inhibit the activation of Yorkie, inasmuch as deletion of Yki reverses the overgrowth phenotypes resulted from loss of Hippo, Warts, Salvador or Mats. Components of the Hippo pathway are highly conserved throughout evolution. The counterparts for the Hippo pathway in Drosophila can all be found in mammals, although they are more diverse and complex [4]. The Hippo orthologs Mst1 and Mst2 utilize the Salvador ortholog WW45/Sav1 to regulate the Warts orthologs Lats1/Lats2. Activated Lats kinases phosphorylate the transcriptional regulators TAZ/YAP (Yorkie orthologs) which promotes 14-3-3 binding to YAP, causing YAP nuclear exit, hereby inhibiting its function. In recent years, increasing numbers of mammalian studies have expanded the large proteins network of the Hippo signaling pathway that controls tissues growth during development and regeneration, as well as in pathological states such as cancer [5].

The upstream regulator of the Hippo pathway and the downstream of Mst1/Mst2 have been diversified considerably in mammals compared with the Drosophila Hippo pathway. Multiple cellular stresses can trigger an adaptive response by activating the Hippo signaling pathway, which may, in turn, maintain the cellular homeostasis. The Hippo/Warts/Mats/Yorkie pathway predicated in Drosophila is not universal in all mammalian tissues in which their regulation and function are different in selected cell types. For examples, Mst1/2 negatively regulates YAP1 in mammalian liver, however, Mst1/2 is not required for YAP1 phosporylation and nuclear exclusion resulted from the cell-cell contact in mouse embryonic fibroblasts(MEFs); Mst1/2 is dispensable for Lats1/2 signaling in MEFs, but not in HeLa cells [6]. Independent of YAP, Mst1 negatively regulates naïve T cell proliferation upon the T cell receptor stimulation, as well as regulates peripheral naïve T cell trafficking and thymus egress [7]. Furthermore, patients with Mst1 deficiency are reported to have a primary immunodeficiency phenotype [8,9]. During the tissues regeneration and tumorigenesis, the Hippo signaling pathway has been shown to cross talk with other signaling players such as Notch and Wnt [10]. Thus, not just for the organ size control, the Hippo pathway receives inputs from multiple extracellular or intracellular signals and interacts with other essential signaling pathways to play critical roles in many aspects for cell fate decisions.

In this issue of the Cell & Bioscience, we have provided some updates on the regulations beyond the canonical Hippo signaling, and their implications in pathological states. Qin et al. will review the recent updates of the roles of Mst1/2 on the cellular redox state regulation, the effects of Mst1/2 deficiency on the development process and tumorigenesis in multiple organs, and their involvement in the immune regulation. The review by Hergovich will summarize the current understanding of mammalian Lats1/2 kinases together with their closest relatives, the NDR1/2 kinases. He will focus on discussion about the regulation of the LATS/NDR family of kinases and their currently known substrates, as well as the biological roles of LATS/NDR kinases. Guo and Zhao follows with a discussion of the function of YAP and TAZ as effectors of cell responses to several extracellular signals including mechanical stress, GPCR signaling, and the Wnt signaling pathway, emphasizing that YAP and TAZ might have different role with cell-type specificity in the promotion of specific cancers. Collectively, these reviews have provided additional information to address the complexity of the hippo signaling pathway in response to physiological signals for regulating cellular and tissues homeostasis.

## References

[B1] Zhao B Tumaneng K Guan KL The Hippo pathway in organ size control, tissue regeneration and stem cell self-renewal Nat Cell Biol 2011 13 8 877 883 10.1038/ncb2303 21808241 PMC3987945

[B2] Pan D The hippo signaling pathway in development and cancer Dev Cell 2010 19 4 491 505 10.1016/j.devcel.2010.09.011 20951342 PMC3124840

[B3] Tapon N Harvey KF The Hippo pathway–from top to bottom and everything in between Semin Cell Dev Biol 2012 23 7 768 769 10.1016/j.semcdb.2012.08.007 22944587

[B4] Avruch J Zhou D Fitamant J Bardeesy N Mou F Barrufet LR Protein kinases of the Hippo pathway: regulation and substrates Semin Cell Dev Biol 2012 23 7 770 784 10.1016/j.semcdb.2012.07.002 22898666 PMC3489012

[B5] Avruch J Zhou D Fitamant J Bardeesy N Mst1/2 signalling to Yap: gatekeeper for liver size and tumour development Br J Cancer 2011 104 1 24 32 10.1038/sj.bjc.6606011 21102585 PMC3039822

[B6] Zhou D Conrad C Xia F Park JS Payer B Yin Y Lauwers GY Thasler W Lee JT Avruch J Mst1 and Mst2 maintain hepatocyte quiescence and suppress hepatocellular carcinoma development through inactivation of the Yap1 oncogene Cancer Cell 2009 16 5 425 438 10.1016/j.ccr.2009.09.026 19878874 PMC3023165

[B7] Mou F Praskova M Xia F Van Buren D Hock H Avruch J Zhou D The Mst1 and Mst2 kinases control activation of rho family GTPases and thymic egress of mature thymocytes J Exp Med 2012 209 4 741 759 10.1084/jem.20111692 22412158 PMC3328371

[B8] Nehme NT Pachlopnik Schmid J Debeurme F Andre-Schmutz I Lim A Nitschke P Rieux-Laucat F Lutz P Picard C Mahlaoui N MST1 mutations in autosomal recessive primary immunodeficiency characterized by defective naive T cells survival Blood 2012 119 15 3458 3468 10.1182/blood-2011-09-378364 22174160 PMC3824282

[B9] Abdollahpour H Appaswamy G Kotlarz D Diestelhorst J Beier R Schaffer AA Gertz EM Schambach A Kreipe HH Pfeifer D The phenotype of human STK4 deficiency Blood 2012 119 15 3450 3457 10.1182/blood-2011-09-378158 22294732 PMC3325036

[B10] Chen L Qin F Deng X Avruch J Zhou D Hippo pathway in intestinal homeostasis and tumorigenesis Protein Cell 2012 3 4 305 310 10.1007/s13238-012-2913-9 22492181 PMC4875478

